# The Mechanism of Gut Microbiota in Breast Cancer Based on the Bulk Transcriptome, Mendelian Randomization Analysis and Single Cell RNA Sequencing

**DOI:** 10.1002/mbo3.70284

**Published:** 2026-04-10

**Authors:** Tianyu Luo, Mengyao Xue, Yi Du, Huiying Chen, Ying Sun, Haidong Sun

**Affiliations:** ^1^ Shenzhen Hospital (Futian) of Guangzhou University of Chinese Medicine Shenzhen Guangdong China; ^2^ Guangzhou University of Chinese Medicine Guangzhou Guangdong China

**Keywords:** biomarkers, breast cancer, gut microbiota, Mendelian randomization, single cell RNA sequencing

## Abstract

Breast cancer (BC) is the leading cause of cancer death in women. Bidirectional regulation between BC and gut microbiota (GM) is established, but GM's mechanistic role in BC pathogenesis remains unclear. Public BC/control samples and GM genome‐wide association study data underwent Mendelian randomization to identify GM‐BC associations and GMRGs. DEGs between BC and controls were analyzed. Candidate genes were derived from intersecting DEGs and GMRGs. Machine learning identified biomarkers, validated by expression analysis. GSEA, immune infiltration, drug screening with molecular docking, and scRNA‐seq were performed. Intersecting 3455 DEGs with GMRGs yielded eight candidates; MCM6 and NR3C1 were validated as biomarkers, enriched in DNA replication pathways. Immune infiltration showed 13 differential immune cells, with macrophages notably influencing biomarkers. Etoposide exhibited strong binding to biomarkers via docking. scRNA‐seq identified epithelial cells as key, with stage‐dependent biomarker expression. This study redefines BC as a microbiome‐regulated network, identifying the MCM6/NR3C1 biomarker pair for early diagnosis and microbiome‐targeted interventions.

## Graphical abstract

This multi‐omics study defines the gut microbiota‐driven MCM6/NR3C1 axis as a key driver of breast cancer, providing robust biomarkers for early diagnosis and actionable therapeutic targets.

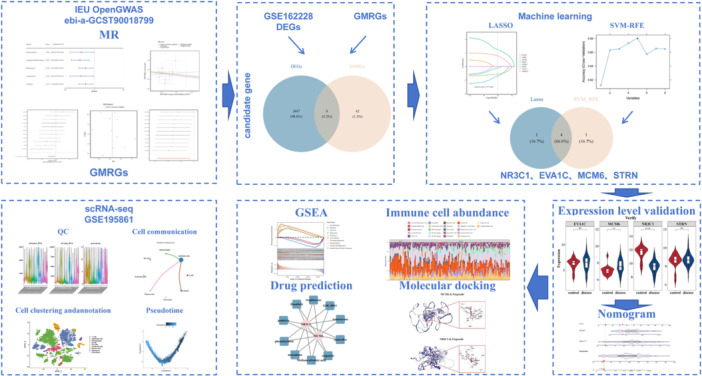

## 1 Introduction

Breast cancer (BC) remains the most frequently diagnosed malignancy in women worldwide, originating from the uncontrolled proliferation of mammary epithelial cells. Over the past four decades, its incidence has risen steadily, and by 2024 BC had become the second most commonly diagnosed cancer across the global population (Siegel et al. 2024). Tumorigenesis is driven by a complex interplay of genetic, environmental, and lifestyle factors (Xiong et al. 2025). Based on molecular subtyping, current therapeutic strategies include surgery, radiotherapy, chemotherapy, endocrine therapy, and targeted agents (Britt et al. 2020). Despite these advances, BC remains associated with substantial morbidity and mortality; approximately 25% of patients eventually develop distant metastases after standard treatment (Dvir et al. 2024). Consequently, identification of novel therapeutic targets and deeper insight into BC biology are urgently needed.

In recent years, mounting evidence has highlighted the pivotal role of the human microbiome in tumorigenesis and tumor progression. Among these microbial communities, as the body's largest micro‐ecosystem, the gut microbiota (GM) orchestrates local immune responses and influences tumor development through the production of bioactive metabolites, modulation of hormone levels, and regulation of systemic inflammatory states (Gori et al. 2019). The GM comprises a dynamic consortium of bacteria, archaea, fungi, viruses, and bacteriophages that colonize the gastrointestinal tract and critically regulate host immunity and metabolic homeostasis (Wang et al. 2023a; Lei et al. 2025). Dysbiosis‐defined as a loss of microbial diversity and overgrowth of pathobionts‐disrupts intestinal barrier integrity, allowing translocation of microbial products and toxins into the circulation, thereby triggering systemic inflammatory responses implicated in diverse pathologies (Sun et al. 2023; Liu et al. 2024; Chen et al. 2024). In oncology, emerging evidence links GM composition to BC progression and treatment response; for instance, bidirectional modulation between GM and chemotherapy‐induced adverse effects has been reported (Corrêa et al. 2022). However, whether specific GM signatures causally influence BC risk remains poorly defined. To gain deeper insight into these mechanisms, this study integrates large‐scale GWAS and multi‐omics approaches. Our goal is to establish causal GM‐BC relationships, decode their molecular circuitry, and identify actionable targets for microbiota‐modulating interventions in BC prevention and therapy.

Mendelian randomization (MR) leverages germline single‐nucleotide polymorphisms (SNPs) as instrumental variables to probe putatively causal links between an exposure and an outcome while circumventing conventional confounding (Yun et al. 2023). By exploiting the random segregation of alleles at conception, MR recapitulates the design of a lifelong randomized controlled trial and has become a mainstay for dissecting causal pathways in complex diseases (Papadopoulou et al. 2023; Yang et al. 2024).

Complementing population‐level MR, single‐cell RNA sequencing (scRNA‐seq) resolves transcriptional heterogeneity at cellular resolution, furnishing an unbiased atlas of cell states and interactions within the tumor microenvironment. This granularity empowers the discovery of previously unrecognized biomarkers and therapeutic targets (Li et al. 2023).

Integrating GWAS and transcriptomic datasets, this study employs MR to dissect the causal relationships between specific gut‐microbiota taxa and BC. Leveraging genes linked to these taxa, this study applies machine‐learning algorithms coupled with rigorous validation to pinpoint robust biomarkers. Subsequent multi‐omics interrogation delineated the associated molecular pathways, immune subpopulations, and druggable targets, while single‐cell profiling identified the critical cell types and tracked dynamic gene‐expression trajectories during cellular differentiation. This “microbiota‐gene‐cell” framework offers a multidimensional perspective on the regulatory networks governing breast‐cancer initiation and progression, laying a theoretical foundation for microbiome‐based prevention and therapeutic strategies.

## 2 Methods

### 2.1 Data Collection

The transcriptome data (GSE162228 and GSE42568) of BC were retrieved from the Gene Expression Omnibus (GEO) database (https://www.ncbi.nlm.nih.gov/geo/). The GSE162228 dataset (GPL570) was the training set, including 109 BC (disease) and 24 control tissue samples. The GSE42568 dataset (GPL570) was the validation set, including 104 BC and 17 control tissue samples. Then, a total of 13 BC and 1 control samples of the scRNA‐seq dataset (GSE195861) were retrieved from the GEO database with the platform being GPL20795. The ebi‐a‐GCST90018799 of BC was retrieved from the Integrative Epidemiology Unit (IEU) Open GWAS database (https://gwas.mrcieu.ac.uk/), with a sample size of 257,730, including 24,133,589 SNPs from 17,389 cases and 240,341 controls of Europeans. The data of GM were downloaded from the GWAS database, including 16S ribosomal RNA gene sequencing profiles and genotyping data of a total of 18,340 participants from 24 cohorts in 11 countries (the United States, South Korea, Canada, Israel, Germany, Denmark, the Netherlands, Finland, Belgium, Sweden, and the United Kingdom).

### 2.2 MR Analysis

To investigate the causal relationship between GM and BC, a MR analysis via the “TwoSampleMR” package (v 0.6.4) (Hemani et al. 2018) was adopted. In MR analysis, GM and BC were regarded as the exposure factors and the outcome, respectively. Then, the instrumental variables (IVs) notably linked to exposure factors (*P* < 5 × 10^−6^) were retained, and the thresholds of linkage disequilibrium (LD) were Clump = TRUE, *R* ^2^ < 0.01, and kb = 100. After that, the number of SNPs was greater than 3, and the SNPs with *F*‐values greater than 10 were reserved. The calculation formula for *F* values was as follows. In the formula, *R* ^2^ represents the cumulative explanatory variance of the selected SNPs, and *N* represents the number of samples.

$$F=\frac{R^{2}\;(n-2)}{1-R^{2}}$$

For instance, for the SNP rs10028567 (beta = −0.092, se = 0.019, *N* = 18,340), the *F*‐statistic was derived as follows: first, t=beta/se≈−4.801; then, the variance explained (*R* ^2^) was estimated using $R^{2}=t^{2}/(t^{2}+N-2)\approx 0.001255$; finally, $F=(N-2)\times [R^{2}/(1-R^{2})]\approx 23.05$. All included SNPs in this study yielded *F* > 10, indicating the absence of weak instrument bias. Finally, the harmonise_data function was utilized to harmonize the effect alleles and effect sizes, and MR Egger (Burgess and Thompson 2017), Weighted median (Bowden et al. 2016), inverse variance weighted (IVW) (Burgess et al. 2015), Simple mode (Chen et al. 2020), and Weighted mode (Hu et al. 2022) were utilized by the mr function to execute MR analysis. Then, the exposure factors with *p* < 0.05 were selected as potential related exposure factors for subsequent analysis based on the results of IVW, while a threshold was not set in the other methods. The results were displayed via the “forestplot” package (v 3.1.3) (Love et al. 2014). Then, the correlation between IVs and BC was shown in the scatter plots via mr_scatter_plot function; the effect size of IVs on outcome was shown in the forest plots; and the symmetry of IVs distribution was displayed in the funnel plots by the mr_funnel_plot function. Furthermore, the sensitivity analyses, including heterogeneity test, pleiotropy test (*p* > 0.05), and leave‐one‐out (LOO) analysis, were executed to evaluate the reliability of MR. The heterogeneity test was evaluated by the mr_heterogeneity function. If the results had heterogeneity (*p* < 0.05), random effects IVW was adopted in the MR; otherwise, fixed effects IVW would be adopted. The pleiotropy test was evaluated by the MR Pleiotropy RESidual Sum and Outlier test (MR‐PRESSO). Lastly, a Steiger test was conducted by the directionality_test function to verify whether the direction of the MR analysis was correct (correct causal direction = TRUE, *p* < 0.05). Most importantly, the GM corresponding to IVs that were obtained through the above analysis was incorporated for subsequent analysis. Finally, the “gprofiler2” package (v 0.6.4) (Kolberg et al. 2020) was utilized to obtain genes corresponding to SNPs of GM, and the GMRGs were obtained by merging and removing duplicate genes.

### 2.3 Identification of DEGs and Candidate Genes

The “limma” package (v 3.54.0) (Love et al. 2014) was utilized to acquire DEGs between BC and control samples (BC vs control) (|log_2_FoldChange (FC)| > 0.5, *p* < 0.05) in all samples of GSE162228. Then, DEGs were visualized by the volcano plot utilizing the “ggplot2” package (v 3.4.4) (Gustavsson et al. 2022), with the names of the top 5 up‐ or down‐regulated genes labeled based on log_2_FC value. Moreover, the top 5 up‐ or down‐regulated genes between 2 groups were also displayed in a heat map. Lastly, the candidate genes were obtained by intersecting the DEGs and GMRGs with the “VennDiagram” package (v 0.1.10) (Chen and Boutros 2011).

### 2.4 Machine Learning and Expression Level Verification

The support vector machine‐recursive feature elimination (SVM‐RFE) and least absolute shrinkage and selection operator (LASSO) logistic regression analyses were conducted, and expression levels of genes were analyzed successively to obtain biomarkers for BC. Firstly, the LASSO and SVM‐RFE algorithms with 10‐fold cross‐validation were conducted via the “glmnet” package (v 4.1‐4) (Yue et al. 2022) and the “e1071” package (v 1.7‐13) (Shi et al. 2023), respectively, in all samples of the GSE162228 dataset. Afterwards, the candidate biomarkers were obtained by the intersection of genes in 2 algorithms via the “VennDiagram” package (v 1.7.3). Then, the expression levels of candidate biomarkers were explored in all samples of the GSE162228 and GSE42568 datasets. The expression differences of genes between BC and control samples were evaluated via the Wilcoxon test (*p* < 0.05). Lastly, the genes with notable differences between BC and control samples and consistent expression trends in 2 datasets were regarded as biomarkers. The correlation analysis among biomarkers was conducted by the Spearman correlation analysis via the “psych” package (v 2.2.9) (Wang et al. 2023b) (|correlation (cor)|) > 0.3, *p* < 0.05). Then, in order to understand the position of biomarkers on chromosomes, the “RCircos” package (v 1.2.2) (Zhang et al. 2013) was used to visualize the position of biomarkers on chromosomes.

### 2.5 Construction of the Nomogram Model

The nomogram model was employed to explore the diagnostic capability of biomarkers for BC. A nomogram was constructed based on biomarkers via the “rms” package (v 6.7‐1) (Xu et al. 2023) in all samples of GSE162228. In the nomogram, the points indicated the individual scores of each biomarker at different values, and the points of each biomarker were added together to obtain the total points. Furthermore, a calibration curve was constructed via the “ResourceSelection” package (v 0.3‐5) (Mcdonald 2013) and the dispersion between predicted and actual values was determined through the Hosmer‐Lemeshow (HL) test to validate the accuracy of the nomogram model (*p* > 0.05). The predictive ability of the nomogram was assessed by a receiver operating characteristic (ROC) curve (area under the curve (AUC) > 0.7) utilizing the “pROC” package (v 1.18.0) (Wang et al. 2023b).

### 2.6 GSEA Analysis

In order to explore the biological functions of biomarkers for BC, the GSEA of each biomarker was performed in all samples of the GSE162228 dataset. Firstly, the “c2.cp.kegg.v7.5.1.symbols.gmt” gene set, which was obtained from the Molecular Signatures Database (MsigDB) (https://www.gsea-msigdb.org/gsea/msigdb/), was utilized as the reference gene set. Then, the Spearman correlation between each biomarker and other genes was calculated by the “psych” package (v 2.2.9) (Wang et al. 2023b). After that, the genes were sorted by their correlation coefficients in descending order. Lastly, the GSEA was performed via the “clusterProfiler” package (v 4.8.3) (|normalized enrichment score| (|NES | ) > 1, *p* < 0.05, false discovery rate (FDR) < 0.25). Finally, the top 10 pathways were displayed via the “enrichplot” package (v 4.10.0) (Zhao et al. 2022) based on the *p* value.

### 2.7 Immune Infiltration Analysis

In this study, the CIBERSORT algorithm was utilized to elucidate the infiltration abundance of the 22 immune cells (Li et al. 2022a) between all BC and control samples in GSE162228. Then, the differential immune cells (DICs) were identified by the Wilcoxon test (*p* < 0.05), and the results were pictured via the ggplot2 package (v 1.18.5). Lastly, the correlation between biomarkers and DICs, as well as among DICs, was performed via the Spearman's correlation analysis by the “psych” package (v 3.4.4) (|cor | > 0.3, *p* < 0.05).

### 2.8 Prediction of Drugs and Molecular Docking

To obtain traditional Chinese medicines associated with biomarkers, the Herb database (http://herb.ac.cn/) was utilized to forecast the Chinese medicine, and the results were drawn using Cytoscape (v 3.10.2) (Shannon et al. 2003). In addition, the drugs associated with biomarkers were applied from the Drug Signatures Database (DSigDB) (https://dsigdb.tanlab.org/DSigDBv1.0/), and the results were pictured by Cytoscape (v 3.10.2). In order to explore the binding ability between biomarkers and corresponding drugs, the molecular docking was performed. Firstly, the 3D chemical structure of non‐toxic drugs with the highest interaction score was acquired from the PubChem database (https://pubchem.ncbi.nlm.nih.gov/). Then, the PDB database (https://www.rcsb.org/) was utilized to acquire the spatial dimensional structure of proteins for biomarkers. Finally, molecular docking between drugs and biomarkers was conducted in the CB‐Dock website (https://cadd.labshare.cn/cb-dock/php/blinddock.php) (binding energy < −5 kcal/mol).

### 2.9 scRNA‐seq Analysis

The scRNA‐seq data were processed utilizing the “Seurat” package (v 5.0.1) (Hao et al. 2021). In detail, the cells with the number of genes in the cell count between 200 and 3000 were reserved (nFeature_RNA represents the number of genes detected in each cell); The genes with expression levels less than 1000 and genes covering at least 3 cells were retained (nCount_RNA represents the total number of genes detected within cells); The proportion of mitochondrial genes in cells was less than 15% (Percent.mt represents the proportion of mitochondrial genes). After that, the NormalizeData function of the “Seurat” package (v 5.1.0) was employed to standardize the feature expression measurement value of each cell by dividing it by the total expression, followed by multiplication with a scale factor of 10,000, and finally taking the logarithm of the result. Afterwards, the top 2000 hypervariable genes in the cells were identified, and the top 10 genes with the greatest variation were marked. Then, the Principal Component Analysis (PCA) of hypervariable genes was conducted by the “runPCA” function. The scree plot was generated based on the PCA results, and the JackStrawPlot function was used to compare the distribution of *p*‐values for each principal component (PC). The number of PCs corresponding to the point where the curve starts to level off was selected for cell clustering (*p* < 0.05). Furthermore, the FindNeighbors and FindClusters functions in the “Seurat” package (v 5.1.0) were utilized to perform clustering analysis for cells, and the results were represented by the RuntSNE function (resolution = 0.4). Finally, the cell annotation was conducted based on the marker genes acquired in the literature (Tokura et al. 2022) and the CellMarker database (http://xteam.xbio.top/CellMarker/), and the expression of marker genes in different cell types was displayed. Specifically, nine cell types were identified using canonical markers: epithelial cells (EPCAM, CDH1, PIP, KRT19/18), macrophages (CD68, CD163, C1QB, MSR1), T cells (CD3D/E/G, CD2), B cells (MS4A1, CD19, CD79A/B), NK cells (NKG7, GNLY, KLRD1), monocytes (S100A8/9, VCAN), fibroblasts (SPARC, COL1A1/3A1, DCN, CALD1), plasma cells (IGHG1/3/4, DERL3), and erythroid cells (HBA1/2, HBB). After that, the functional enrichment on cells was analyzed by the “ReactomeGSA” package (v 1.12.0) (Grentner et al. 2024).

To determine the key cells of BC in the GSE176078 dataset, the differential expressions of biomarkers in different cells were compared (*p* < 0.05), and the cells with differential expression of biomarkers were selected as the key cells. To reclassify key cells into different clusters, the same methods mentioned earlier were utilized. Then, the pseudo‐temporal analysis of key cells was achieved through the “Monocle” package (v 2.26.0) (Cao et al. 2019), and the expression of biomarkers at different periods of key cells was displayed by the plot_pseudo‐time_heatmap function. In addition, in order to explore the interactions and communication between cells, the “CellChat” package (v 1.6.1) (Jin et al. 2025) was utilized to analyze the communication network between the cells. Afterwards, the correlation between receptors and ligand genes, and biomarkers was analyzed utilizing the previously mentioned methods. These receptor and ligand genes are the genes that undergo significant changes between epithelial cells and other cells in the communication network.

### 2.10 Statistical Analysis

Bioinformatics analyses were conducted via the R programming language(v 4.3.1). The Wilcoxon test was utilized to compare the differences between 2 groups. *p* < 0.05 was considered statistically significant.

## 3 Results

### 3.1 Identification of GMRGs

**Figure 1:**
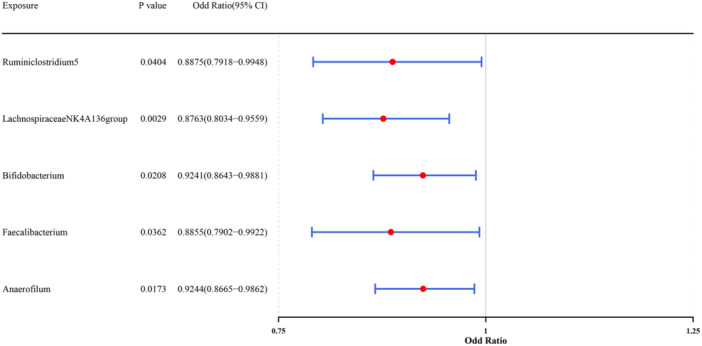
Mendelian randomization analysis of gut microbiota and breast cancer (BC) Mendelian randomization (MR) analysis identified five gut microbiota taxa with significant causal relationships with BC based on inverse variance weighted (IVW) method (*p* < 0.05). The forest plot displays odds ratios (ORs) and 95% confidence intervals (CIs) for the association between each gut microbiota taxon and BC risk. Ruminiclostridium5 (OR = 0.8875, 95% CI: 0.7918–0.9948), Lachnospiraceae NK4A136 group (OR = 0.8763, 95% CI: 0.8034–0.9559), Bifidobacterium (OR = 0.9241, 95% CI: 0.8643–0.9881), Faecalibacterium (OR = 0.8855, 95% CI: 0.7902–0.9922), and Anaerofilum (OR = 0.9244, 95% CI: 0.8665–0.9862) were identified as protective factors against BC. Error bars represent 95% CIs. BC, breast cancer; GM, gut microbiota; IVW, inverse variance weighted.

In MR analysis, only 5 GM that had significant causal relationships with BC were obtained based on IVW results (*p* < 0.05) (Figure 1). The scatter plot indicated that the *Ruminiclostridium5* (OR = 0.8875, 95%CI: 0.7918–0.9948), *LachnospiraceaeNK4A136group* (OR = 0.8763, 95%CI: 0.8034–0.9559), *Bifidobacterium* (OR = 0.9241, 95%CI: 0.8643–0.9881), *Faecalibacterium* (OR = 0.8855, 95%CI: 0.7902–0.9922), and *Anaerofilum* (OR = 0.9244, 95%CI: 0.8665–0.9862) were protective factors for BC (Supporting Information Figure 1). In addition, the forest map showed the same results as the scatter plot (Supporting Information Figure 2). The funnel plot showed that the distribution of SNPs in GM was generally symmetrical and evenly distributed, indicating that the selected IVs conformed to Mendel's second law (Supporting Information Figure 3). In the heterogeneity test, the *p* values of 5 GM were greater than 0.05, indicating that there was no heterogeneity (Table 1). Furthermore, in the horizontal pleiotropy test, the *p* values and presso *p* values of 5 GM were greater than 0.05, indicating that there was no horizontal pleiotropy in the results (Table 2). The results of LOO showed that when each SNP was gradually removed, the remaining SNPs had little effect on the outcome, indicating that the MR results were reliable (Supporting Figure 4). The Steiger test results indicated that these 5 GM, including *Ruminiclostridium5*, *LachnospiraceaeNK4A136group*, *Bifidobacterium*, *Faecalibacterium*, and *Anaerofilum*, had unidirectional causal relationships with BC (Table 3). Therefore, these 5 GM that complied with the Steiger test were considered for inclusion in the subsequent study. Finally, a total of 50 GMRGs were obtained (Supporting Information Table 1).

**Table 1:** The results of heterogeneity test.

Exposure	Method	Q_pval
genus.Ruminiclostridium5.id.11355	Inverse variance weighted	0.4829
genus.LachnospiraceaeNK4A136group.id.11319	Inverse variance weighted	0.8980
genus.Bifidobacterium.id.436	Inverse variance weighted	0.2848
genus.Faecalibacterium.id.2057	Inverse variance weighted	0.3569
genus.Anaerofilum.id.2053	Inverse variance weighted	0.5425

**Table 2:** The results of horizontal pleiotropy test.

Exposure	*p*val	presso_*p*val
genus.Ruminiclostridium5.id.11355	0.713	0.338
genus.LachnospiraceaeNK4A136group. id.11319	0.864	0.936
genus.Bifidobacterium.id.436	0.436	0.249
genus.Faecalibacterium.id.2057	0.442	0.262
genus.Anaerofilum.id.2053	0.520	0.191

**Table 3:** The resuts of steiger test.

Exposure	Correct_causal_direction	Steiger_*p*val	Type
genus.Anaerofilum.id.2053	TRUE	1.183E‐49	genus.Anaerofilum.id.2053
genus.Bifidobacterium.id.436	TRUE	1.213E‐187	genus.Bifidobacterium.id.436
genus.Faecalibacterium.id.2057	TRUE	8.355E‐46	genus.Faecalibacterium.id.2057
genus.LachnospiraceaeNK4A136group.id.11319	TRUE	1.995E‐64	genus.LachnospiraceaeNK4A136group.id.11319
genus.Ruminiclostridium5.id.11355	TRUE	4.421E‐60	genus.Ruminiclostridium5.id.11355

### 3.2 Identification of Biomarkers

**Figure 2:**
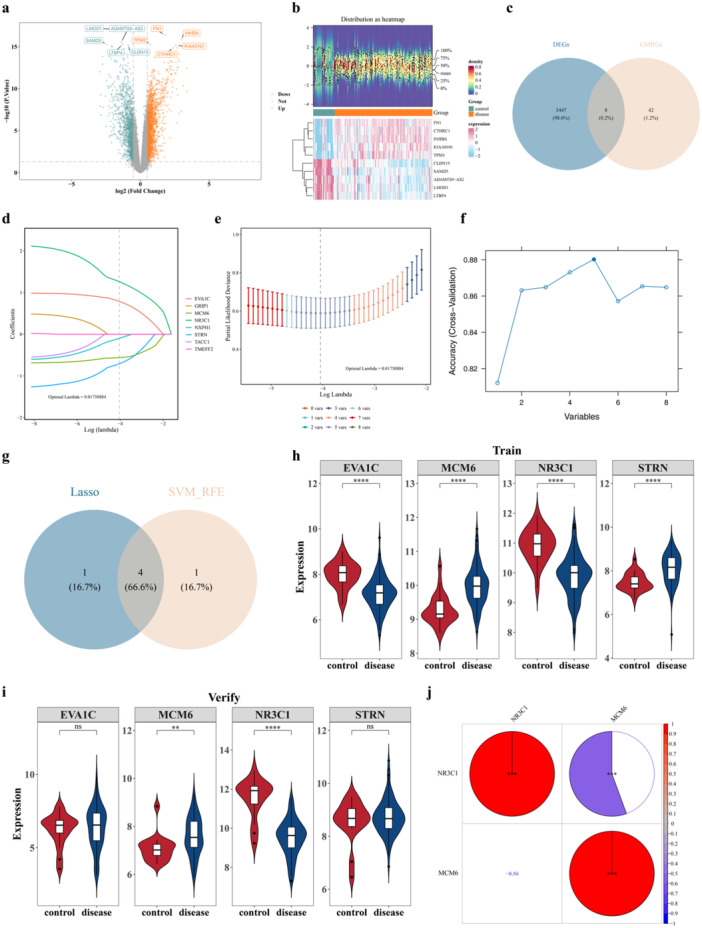
Identification of biomarkers MCM6 and NR3C1. (a) Volcano plot showing differentially expressed genes (DEGs) between breast cancer (BC) and control samples in the GSE162228 dataset (|log2FC | > 0.5, *p* < 0.05). Red dots represent upregulated genes, blue dots represent downregulated genes, and gray dots represent non‐significant genes. (b) Heatmap displaying the top five upregulated and downregulated genes between BC and control samples. (c) Venn diagram showing the intersection of 3455 DEGs and 50 gut microbiota‐related genes (GMRGs), yielding eight candidate genes. (d) LASSO coefficient profiles of the eight candidate genes; vertical line represents the optimal lambda value (lambda.min = 0.01750884). (e) LASSO cross‐validation curve; error bars represent standard error. (f) SVM‐RFE ranking of candidate genes; lower rank indicates higher importance. (g) Venn diagram showing the intersection of genes identified by LASSO and SVM‐RFE algorithms, yielding four candidate biomarkers. (h) Expression levels of MCM6 in BC and control samples across two datasets (GSE162228 and GSE42568). (i) Expression levels of NR3C1 in BC and control samples across two datasets. (j) Spearman correlation analysis revealed a correlation between MCM6 and NR3C1 (cor = −0.56, *p* < 0.001). Data are presented as mean ± SEM. *p < 0.05, ***p < 0.001.

In the GSE162228 dataset, a total of 3,455 DEGs were determined, of which 2061 genes were up‐regulated in the BC group (Figure 2A,B). Then, eight candidate genes (NR3C1, NXPH1, EVA1C, TMEFF2, MCM6, STRN, TACC1, and GRIP1) were obtained through the intersection of DEGs and GMRGs (Figure 2C). After that, a total of five genes (NR3C1, NXPH1, EVA1C, MCM6, and STRN) were obtained utilizing the LASSO algorithm (lambda.min = 0.01750884) (Figures 2D,E), and 5 genes were selected in the SVM‐RFE algorithm (NR3C1, TACC1, EVA1C, MCM6, and STRN) (Figure 2F). Then, a total of 4 candidate biomarkers (NR3C1, EVA1C, MCM6, and STRN) were obtained from the intersection gene set of the two algorithms (Figure 2G). Finally, only MCM6 and NR3C1 showed notable differences between BC and control samples (*p* < 0.05). NR3C1 showed a downward trend while MCM6 showed an upward trend in the BC group of both datasets (Figure 2H,I). Therefore, MCM6 and NR3C1 were regarded as the biomarkers. Then, the correlation results showed that MCM6 was notably negatively correlated with NR3C1(cor = −0.56, *p* < 0.001) (Figure 2J).

### 3.3 High Accuracy of the Nomogram

**Figure 3:**
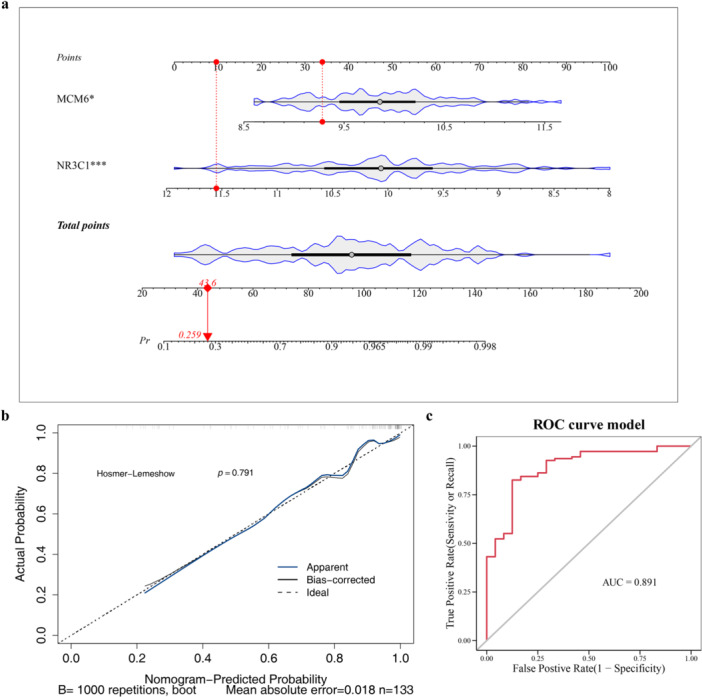
Nomogram model for breast cancer (BC) diagnosis. (a) Nomogram constructed based on biomarkers MCM6 and NR3C1 for predicting BC risk. Points indicate individual scores for each biomarker at different values; total points are calculated by summing individual scores. (b) Calibration curve showing the agreement between predicted and actual probabilities of BC; the slope is close to 1, and the Hosmer‐Lemeshow test *p* = 0.791. (c) Receiver operating characteristic (ROC) curve demonstrating the diagnostic performance of the nomogram model; area under the curve (AUC) = 0.891.

In the nomogram model, the higher the total points, the higher the risk of BC. The results showed that MCM6 and NR3C1 had good diagnostic ability for BC, especially NR3C1 (Figure 3A). The slope of calibration curves was close to 1, and the *p* value was greater than 0.05 (*p* = 0.791), indicating that the nomogram had good accuracy in predicting BC (Figure 3B). In addition, the AUC of the ROC curve was greater than 0.7 (AUC = 0.891), indicating that the model had commendable diagnostic value for BC patients (Figure 3C). These results indicated that the nomogram model had outstanding predictive ability for BC patients.

### 3.4 Enrichment Pathway and Chromosome Localization of Biomarkers

**Figure 4:**
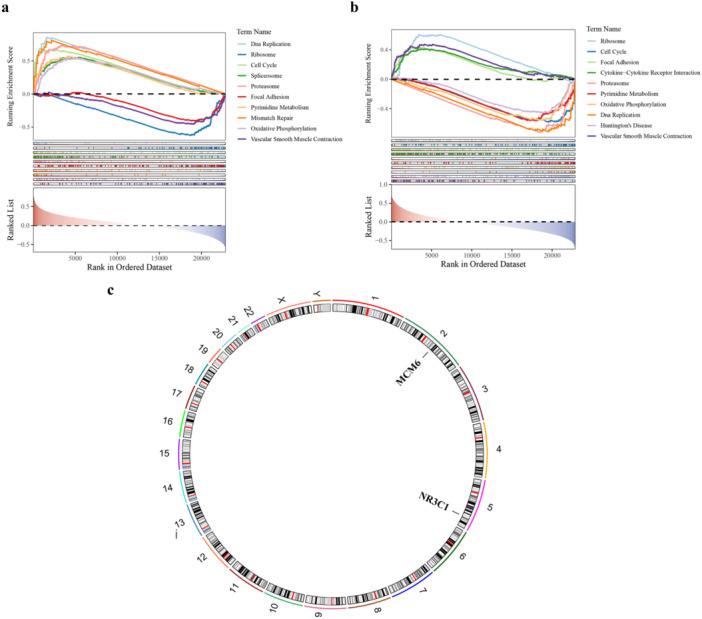
Enrichment pathways and chromosome localization of biomarkers. (a) GSEA showing enrichment of MCM6 in the DNA replication pathway (normalized enrichment score [NES] = 1.82, *p* < 0.05, false discovery rate [FDR] < 0.25). The top panel shows the running enrichment score, the middle panel shows the positions of gene set members in the ranked list, and the bottom panel shows the ranked list of genes. (b) GSEA showing enrichment of NR3C1 in proteasome pathway (NES = 1.75, *p* < 0.05, FDR < 0.25). (c) Chromosome localization of biomarkers MCM6 and NR3C1. MCM6 is located on chromosome 2 (2p12), while NR3C1 is located on chromosome 5 (5q31.3). The color intensity represents the expression level of the genes. RCircos visualization shows the positions of the biomarkers on their respective chromosomes.

The GSEA results showed that the number of notable pathways enriched by MCM6 and NR3C1 was 89 and 82, respectively. To enhance clarity, the top 10 enriched pathways are visualized in Figure 4A,B, while the comprehensive analysis results are provided in Supporting Information Tables 2 and 3. Specifically, MCM6 and NR3C1 were both enriched in DNA replication, proteasome, ribosome, etc, indicating that biomarkers most likely affected the development of BC through these notably enriched pathways. The chromosome localization results indicated that 2 biomarkers were located on different chromosomes, with MCM6 on chromosome 2 and NR3C1 on chromosome 5 (Figure 4C).

### 3.5 Immune Cells Linked to Biomarkers in BC

**Figure 5:**
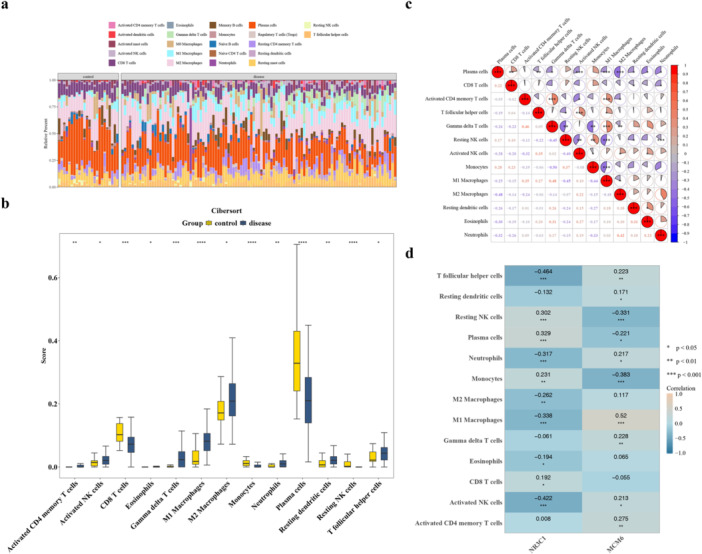
Immune cell infiltration analysis in BC. (a) Bar plot showing the relative abundance of 22 immune cell types in BC and control samples. (b) Heatmap displaying 13 differential immune cells between BC and control samples (*p* < 0.05), including CD8 + T cells, activated NK cells, and macrophages. (c) Correlation matrix among DICs; red indicates positive correlation, blue indicates negative correlation; M1 macrophages positively correlated with gamma delta T cells (cor = 0.480, *p* < 0.001), and M2 macrophages negatively correlated with plasma cells (cor = −0.480, *p* < 0.001). (d) Correlation between biomarkers and M1 macrophages; MCM6 showed a significant positive correlation with M1 macrophages (cor = 0.520, *p* < 0.001), while NR3C1 showed a significant negative correlation with M1 macrophages (cor = −0.338, *p* < 0.001). Data are presented as mean ± SEM. *p < 0.05, ***p < 0.001.

The abundance of 22 types of immune cells between BC and control samples was shown in Figure 5A. In addition, a total of 13 DICs, such as CD8 T cells and activated natural killer (NK) cells, were obtained in this study (*p* < 0.05) (Figure 5B, Supporting Information Table 4). The correlation analysis among the DICs indicated that M1 macrophages were positively correlated with gamma delta T cells (cor = 0.480, *p* < 0.001), and M2 macrophages were negatively correlated with plasma cells (cor = −0.480, *p* < 0.001) (Figure 5C). MCM6 (cor = 0.520, *p* < 0.001) had notably positive correlation with M1 macrophages while NR3C1 (cor = −0.338, *p* < 0.001) had notably negative correlation with M1 macrophages (Figure 5D). These results showed that these DICs, especially macrophages, might have a notable impact on biomarkers in BC.

### 3.6 The Drugs Related to Biomarkers

**Figure 6:**
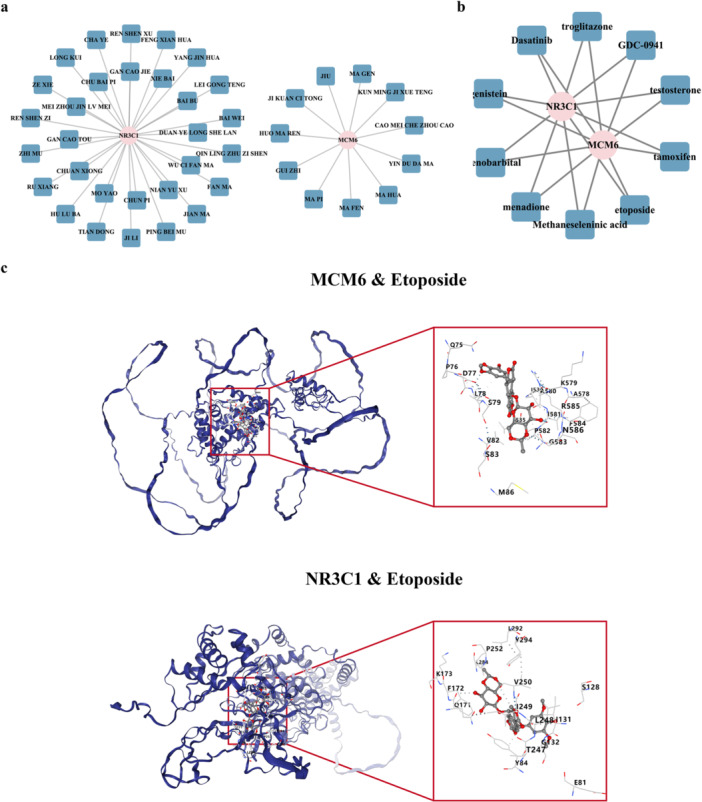
Drug prediction and molecular docking analysis. (a) Network visualization showing 30 traditional Chinese medicines associated with NR3C1 and 11 traditional Chinese medicines associated with MCM6. Nodes represent drugs and biomarkers; edges represent associations. (b) Network visualization showing 10 drugs associated with both NR3C1 and MCM6, including etoposide and testosterone. (c) Molecular docking results demonstrating the binding modes between etoposide and biomarkers; etoposide showed strong binding affinity with MCM6 (binding energy = −8.50 kcal/mol) and NR3C1 (binding energy = −9.50 kcal/mol). The left panel shows the 3D binding conformation, and the right panel shows the 2D ligand interaction diagram. Hydrogen bonds are indicated by green dashed lines.

A total of 30 traditional Chinese medicines, such as baiwei, were found to be associated with NR3C1, and 11 traditional Chinese medicines, such as guizhi, were found to be associated with MCM6 (Figure 6A). In addition, a total of 10 drugs, such as etoposide and testosterone, were found to be associated with NR3C1 and MCM6 (Figure 6B). The molecular docking results showed that etoposide had a good binding mode with MCM6 (binding energy = −8.50 kcal/mol) and NR3C1 (binding energy = −9.50 kcal/mol) (Figure 6C, Table 4). The above results revealed that these drugs associated with biomarkers, especially etoposide, could have potential therapeutic effects on BC patients.

**Table 4:** The binding energy between biomarkers and drugs.

Gene	AlphaFoldDB ID	DRUG	PubChem CID	Binding energy (kcal/mol)
MCM6	AF‐Q14566‐F1‐v4	Etoposide	36462	−8.5
NR3C1	AF‐P04150‐F1‐v4	Etoposide	36462	−9.5

### 3.7 Identification of Key Cells

**Figure 7:**
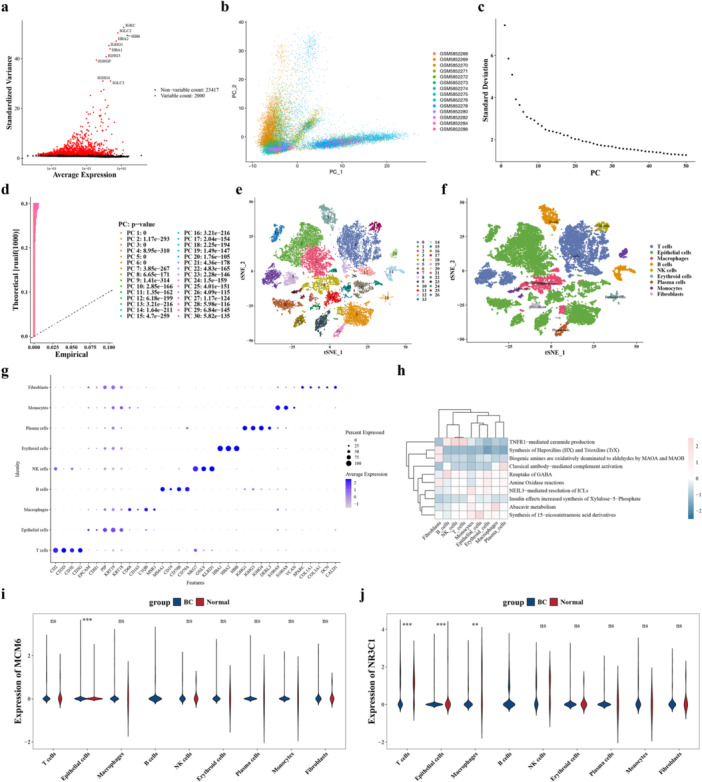
Single‐cell RNA sequencing analysis and identification of key cells. (a) Feature plot showing the top 2000 hypervariable genes used for principal component analysis (PCA). (b) PCA plot revealing that the distribution of cells across different samples is relatively centered without batch effects. (c) Elbow plot showing the proportion of variance explained by each principal component (PC); the top 30 PCs were selected for downstream analysis. (d) JackStraw plot comparing the distribution of P‐values for each PC; PCs corresponding to the point where the curve starts to level off were selected (*p* < 0.05). (e) UMAP plot visualizing 27 cell clusters identified from 22,967 cells. (f) UMAP plot annotated with nine cell types based on marker gene expression: monocytes, T cells, epithelial cells, B cells, NK cells, macrophages, fibroblasts, plasma cells, and erythroid cells. (g) Feature plots showing the expression of marker genes in different cell types; most marker genes exhibited high levels of specific expression in their corresponding cells. (h) Bubble plot displaying functional enrichment results for the nine cell types; pathways include abacavir metabolism, etc. (i, j). Violin plots showing the expression of biomarkers in nine cell types; MCM6 (i) and NR3C1 (j) showed significant differences in epithelial cells between breast cancer and control samples (*p* < 0.05). Data are presented as mean ± SEM. *p < 0.05.

A total of 22,967 cells and 25,417 genes were retained for further analysis after undergoing quality control (Supporting Information Figure 5A,B). Then, the top 2000 hypervariable genes, such as IGHG3, were designated for PCA (Figure 7A). The PCA results revealed that the distribution of cells in different samples was relatively centered without a batch effect (Figure 7B). Afterwards, the top 30 PCs were selected, and all cells were divided into 27 clusters (Figure 7C–E). Then, a total of nine cells (monocytes, T cells, epithelial cells, B cells, NK cells, macrophages, fibroblasts, plasma cells, and erythroid cells) were obtained based on the expression of marker genes in different cell clusters (Figure 7F). The expression of marker genes in different cells revealed that most marker genes had high levels of specific expression in their corresponding cells (Figure 7G). The results of functional enrichment displayed that the significant pathways enriched by the nine cells included abacavir metabolism, etc (Figure 7H). Then, the expression of biomarkers in the nine cells indicated that MCM6 and NR3C1 showed notable differences in the epithelial cells between BC and control samples (*p* < 0.05) (Figure 7I,J). So epithelial cells were selected as the key cells in this study.

### 3.8 Exploration of Epithelial Cells

**Figure 8:**
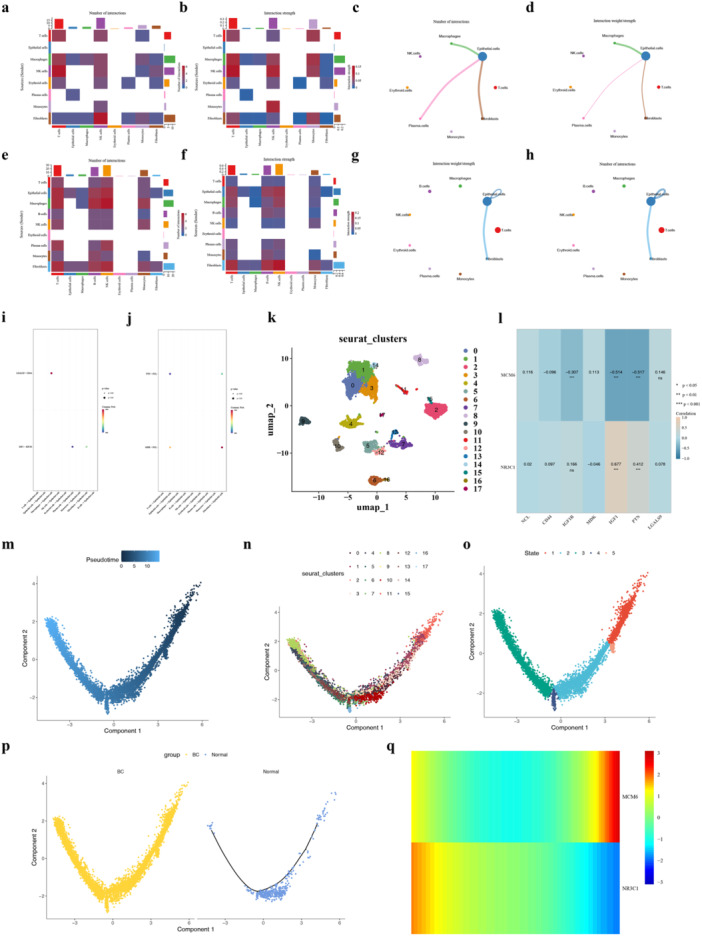
Exploration of epithelial cells in breast cancer (BC). (a–d) Cell–cell communication networks in control samples showing interactions between epithelial cells and macrophages (a), fibroblasts (b), plasma cells (c), and other cell types (d). (e–h) Cell–cell communication networks in BC samples showing interactions between epithelial cells and fibroblasts (e), with no significant interactions with macrophages (f) or plasma cells (g, h). (i) Strong LGALS9‐CD44 interaction between epithelial cells and macrophages in control samples. (j) Strong MDK‐NCL interaction between epithelial cells and fibroblasts in BC samples. (k) Correlation analysis showed MCM6 had a significant negative correlation with PTN (cor = −0.517, *p* < 0.001), while NR3C1 had a significant positive correlation with IGF1 (cor = 0.677, *p* < 0.001). (l) UMAP plot showing epithelial cells reclustered into 18 cell clusters. (m, n) Feature plots showing cluster distribution in early (m) and late (n) differentiation stages; cluster 8 predominated in early stages, while cluster 11 predominated in late stages. (o, p) Pseudotime trajectory analysis showing epithelial cell differentiation divided into 5 stages; the 18 cell clusters were distributed throughout the differentiation process in the BC group (o), with specific cluster distribution across stages (p). (q) Heatmap showing dynamic expression patterns of biomarkers during epithelial cell differentiation; NR3C1 expression gradually decreased, while MCM6 expression peaked in the late differentiation stage. Data are presented as mean ± SEM. ***p < 0.001.

Analysis of cell communication networks among the nine cell types revealed distinct interaction patterns. In control samples, epithelial cells interacted with macrophages, fibroblasts, and plasma cells (Figure 8A–D). However, in BC samples, they interacted exclusively with fibroblasts (Figure 8E–H). In detail, a strong interaction of LGALS9‐CD44 between epithelial cells (Figure 8I) and macrophages appeared in control samples, and a strong interaction of MDK‐NCL between epithelial cells and fibroblasts appeared in BC samples (Figure 8J). MCM6 (cor = −0.517, *p* < 0.001) had notably negative correlation with PTN while NR3C1 (cor = 0.677, *p* < 0.001) had notably positive association with IGF1 (Figure 8K). Then, epithelial cells were reclustered into 18 cell clusters (Figure 8L). In the early stage, cluster 8 was the main cluster, and in the final stage, cluster 11 was the main cluster (Figure 8M,N). The results showed that differentiation of epithelial cells was divided into five stages, and the 18 cell clusters were distributed throughout the differentiation process in the BC group (Figure 8O,P). The expression level of NR3C1 gradually decreased during the differentiation process, and the expression level of MCM6 was highest in the late stage of epithelial cell differentiation (Figure 8Q). These results revealed that the development of epithelial cells might be associated with the expression of two biomarkers.

## 4 Discussion

BC remains the most prevalent malignancy among women globally, posing a significant public health challenge due to its increasing incidence and complex pathogenesis. The GM modulates the immune system in BC patients through multiple mechanisms: it regulates the proliferation and differentiation of regulatory T cells, promotes the expression of secretory immunoglobulin A, and modulates neutrophil production (Kang et al. 2025). An integrative multi‐omics approach combining MR analysis, bulk transcriptome profiling, and single‐cell RNA sequencing was employed to elucidate the causal relationship between GM and BC development. Through rigorous MR analysis, we identified several microbial taxa with protective effects against BC, including *Ruminiclostridium5*, *Lachnospiraceae* NK4A136 group, *Bifidobacterium*, *Faecalibacterium*, and *Anaerofilum*. Subsequently, 50 GMRGs were derived from these taxa. Notably, our MR analysis exclusively identified protective GM taxa without any risk‐associated taxa. This finding may reflect a biological reality where specific beneficial microbes are more robustly linked to the modulation of BC risk within the current GWAS datasets. Alternatively, it might be attributed to the stringent statistical thresholds and the inherent power of the available microbiome GWAS data, which may prioritize the identification of consistent protective associations. By integrating these with differentially expressed genes (DEGs) from BC datasets, eight candidate genes were obtained. From this pool, MCM6 and NR3C1 were selected as robust BC biomarkers through machine learning algorithms and cross‐dataset validation. Comprehensive functional analysis revealed that these biomarkers converge on critical pathways, including DNA replication, proteasome, and ribosome signaling, with significant correlations to immune cell populations, particularly macrophages. Molecular docking further demonstrated the strong binding affinity of etoposide to both biomarkers. Epithelial cells were pinpointed as the critical cell type in BC pathogenesis through single‐cell analysis, with distinctive expression patterns of the biomarkers during cellular differentiation. Collectively, a novel “microbiota‐gene‐cell” framework was established that elucidates GM‐mediated molecular mechanisms in BC and identifies potential therapeutic targets.

The identification of MCM6 and NR3C1 as pivotal BC biomarkers provides significant mechanistic insights into tumor pathogenesis. MCM6 is a member of the minichromosome maintenance (MCM) protein family. It functions as a highly conserved DNA helicase that is essential for replication initiation and genome duplication (Wang et al. 2022; Gao et al. 2023). It was revealed that MCM6 is significantly enriched in DNA replication, proteasome, and ribosome pathways, aligning with its core function as a DNA helicase, whereas its involvement in proteasome and ribosome pathways reflects broad regulatory roles in cellular proliferation (Shekhar et al. 2023). The upregulation of MCM6 in BC was found to be consistent with previous reports demonstrating MCM6 overexpression in gastrointestinal cancers, neuroblastoma, and BC, where it promotes tumor proliferation and progression (Wang et al. 2022). The dysregulation of DNA replication is considered a hallmark of cancer, where impaired replication machinery leads to increased spontaneous DNA damage and genomic instability (Gu et al. 2023). Cancer cells further exacerbate this instability through elevated metabolic activity, generating high levels of reactive oxygen species (ROS) that induce DNA damage. Elevated MCM6 expression promotes BC tumorigenesis by sustaining DNA replication fidelity, whereas its depletion or K599 crotonylation induces replication stress and DNA damage ‐ thereby suppressing cancer cell proliferation and enhancing chemosensitivity. This process is dynamically regulated by SIRT7 and counterbalanced by RNF8‐mediated ubiquitination (BC) (Song et al. 2025). This suggests that MCM6 may serve as a specific biomarker for predicting poor prognosis in BC, and its inhibition could represent a viable therapeutic strategy. Building upon these findings, we propose a “dual‐hit” model where MCM6 and NR3C1 synergistically drive BC progression. The overexpression of MCM6 accelerates DNA replication and cell proliferation, while the downregulation of NR3C1 impairs tumor‐suppressive glucocorticoid signaling and apoptosis. This combination of enhanced proliferative drive and loss of inhibitory regulation creates a cellular environment highly conducive to rapid tumor growth and genomic instability.

NR3C1, encoding the glucocorticoid receptor, has been reported to play a critical role in regulating inflammatory responses and immune homeostasis. NR3C1 serves as the terminal effector of the hypothalamic‐pituitary‐adrenal (HPA) axis, mediating both genomic and non‐genomic actions of glucocorticoids. Its downregulation is implicated in cancer‐associated HPA axis dysregulation, particularly in stress response pathways (Park et al. 2016). Downregulation of NR3C1 in BC was observed, which may reflect dysregulation of the HPA axis commonly seen in cancer patients. The HPA axis and inflammatory processes are known to engage in bidirectional communication, and disturbances in this system can lead to glucocorticoid resistance and elevated pro‐inflammatory cytokines (Shim et al. 2022). NR3C1 is upregulated by MDK‐mediated NF‐κB activation to promote BC cell proliferation and migration; conversely, NR3C1 silencing suppresses proliferation, migration, and epithelial‐mesenchymal transition (Zhang et al. 2022). In luminal A and basal‐like BC subtypes, NR3C1 dysregulation modulates tumor cell proliferation characteristics through pathway‐specific mechanisms (Xiong et al. 2022). This apparent discrepancy may reflect the complex regulatory network of NR3C1 in BC pathogenesis, potentially influenced by tumor microenvironment heterogeneity or molecular subtypes. The convergence of both biomarkers on DNA replication pathways suggests a potential synergistic mechanism. MCM6 upregulation drives uncontrolled proliferation, while NR3C1 downregulation compromises genomic stability, collectively facilitating BC development.

Thirteen differentially abundant immune cell types between BC and control samples were revealed through immune infiltration analysis, with macrophages exhibiting particularly significant correlations with the identified biomarkers. Notably, a strong positive correlation with M1 macrophages (cor = 0.520, *p* < 0.001) was demonstrated by MCM6, while NR3C1 showed a significant negative correlation with the same cell population (cor = −0.338, *p* < 0.001). This differential relationship provides valuable insights into the complex interplay between tumor cells and the immune microenvironment, which may also be shaped by GM‐derived metabolites that regulate immune homeostasis and macrophage function (Ren et al. 2025). Tumor‐associated macrophages respond to the stiff fibrotic microenvironment by initiating collagen biosynthesis via TGF‐β. This process inadvertently creates a metabolically unfavorable environment, suppressing effective CD8+ T cell antitumor responses (Xiao and Li 2025). This process contributes to the characteristic fibrosis observed during tumor progression, which involves excessive extracellular matrix accumulation and correlates with diminished anti‐tumor immune infiltration (Tharp et al. 2024).

Emerging research has revealed the pro‐tumorigenic functions of M1 macrophages in specific tumor microenvironments, demonstrating a complex bidirectional role. Studies indicate that M1‐like tumor‐associated macrophages not only fail to suppress tumor progression in certain cancer types but may actually promote tumor advancement and metastasis (Boutilier and Elsawa 2021).

CD68+ HLA‐DR + M1‐like tumor‐associated macrophages have been shown to increase tumor cell motility in hepatocellular carcinoma via the NF‐κB/FAK pathway. Similarly, in oral squamous cell carcinoma, tumor‐derived exosomes have been found to induce TAM conversion toward an M1‐like phenotype, ultimately facilitating malignant tumor dissemination (Xiao et al. 2018; Wang et al. 2014). The pro‐metastatic role of M1‐like macrophages may be partially attributed to their secretion of inflammatory cytokines, including IL‐1β, TNF‐α, and IL‐6, which promote vasoproliferation through both direct and indirect mechanisms (Nakao et al. 2005; Fujimoto et al. 2002).

This work found that 13 differentially abundant immune cell types between BC and control samples were revealed through immune infiltration analysis, with macrophages exhibiting particularly significant correlations with the identified biomarkers. Notably, a strong positive correlation with M1 macrophages (cor = 0.520, *p* < 0.001) was demonstrated by MCM6, while NR3C1 showed a significant negative correlation with the same cell population (cor = −0.338, *p* < 0.001). This differential relationship provides valuable insights into the complex interplay between tumor cells and the immune microenvironment. While M1‐like TAMs are traditionally recognized for their anti‐tumor potential, recent evidence and our findings suggest their role may be context‐dependent (Wan et al. 2025; Xia et al. 2025). It is suggested that MCM6 is associated with an altered immune landscape featuring M1 macrophage infiltration, while NR3C1 downregulation might impair the regulatory mechanisms that normally constrain excessive inflammatory responses. Rather than a purely pro‐tumorigenic or anti‐tumorigenic effect, this correlation likely reflects a sophisticated immune remodeling process. This dual mechanism could contribute to the establishment of a complex microenvironment that potentially facilitates tumor immune evasion under specific conditions. The correlation between biomarkers and specific immune cell populations further underscores the potential of MCM6 and NR3C1 as indicators of immunological status in BC, which could inform personalized immunotherapy strategies.

Significant therapeutic potential for targeting the identified biomarkers was revealed through drug prediction and molecular docking analyses. Notably, the strong binding affinity of etoposide to both MCM6 (binding energy = −8.50 kcal/mol) and NR3C1 (binding energy = −9.50 kcal/mol) was demonstrated, suggesting its potential utility in BC treatment. Etoposide, a topoisomerase II inhibitor, functions by inducing DNA double‐strand breaks during replication, which aligns mechanistically with the DNA replication pathway enrichment observed for MCM6. It is demonstrated that etoposide exhibits significant antitumor effects in BC cell lines, particularly when combined with γ‐tocotrienol (Idriss et al. 2022). Additionally, etoposide has been shown to have clinical efficacy in triple‐negative BC, a particularly aggressive BC subtype (Zhang et al. 2020). Thirty traditional Chinese medicines associated with NR3C1, including baiwei (Cynanchum atratum), and 11 associated with MCM6, such as guizhi (Cinnamomum cassia), were identified. Of particular interest, compounds derived from guizhi, notably cinnamaldehyde, have been shown to inhibit BC cell proliferation and migration (Wu et al. 2022), providing preliminary validation for the computational predictions. These findings collectively suggest that etoposide and specific traditional medicines may represent promising therapeutic options for BC patients, potentially through modulation of MCM6 and NR3C1 expression or activity. Future studies should investigate the efficacy of these compounds in preclinical BC models and explore potential synergistic effects in combination therapies.

Unprecedented resolution of cellular heterogeneity in BC was provided through single‐cell RNA sequencing analysis, with epithelial cells being identified as the critical cell type associated with the biomarkers. Epithelial cells serve as the cellular origin of BC, and their abnormal proliferation and differentiation are recognized as critical events in tumor initiation and progression (Herbein and El Baba 2024). Notably, distinct interaction patterns between epithelial cells and other cell populations in BC versus control samples were revealed through cell‐cell communication analysis. In control tissues, epithelial cells exhibited communication with macrophages, fibroblasts, and plasma cells, whereas in BC samples, interactions were limited primarily to fibroblasts. Specifically, the LGALS9‐CD44 interaction between epithelial cells and macrophages observed in control samples was replaced by MDK‐NCL interactions between epithelial cells and fibroblasts in BC tissues. This shift in communication networks suggests a fundamental reprogramming of the tumor microenvironment during BC development.

Distinctive biomarker expression patterns throughout the differentiation trajectory were revealed through pseudotemporal analysis. NR3C1 expression was found to progressively decrease during epithelial cell differentiation, while MCM6 expression peaked in the late differentiation stages (Li et al. 2022b). This dynamic expression pattern aligns with the established roles of these genes in cell cycle regulation and differentiation processes. It is suggested that MCM6 may drive uncontrolled proliferation of epithelial cells through regulation of cell cycle progression and DNA replication (Zeng et al. 2021), while NR3C1 downregulation may compromise the normal differentiation program, contributing to the maintenance of a progenitor‐like state conducive to tumorigenesis (Xiong et al. 2022). The correlation between biomarkers and specific ligand‐receptor pairs (MCM6 with PTN, NR3C1 with IGF1) further elucidates potential mechanistic links between these biomarkers and cellular communication pathways in BC. These single‐cell level insights provide compelling evidence supporting the biological relevance of MCM6 and NR3C1 in BC pathogenesis and establish a foundation for developing cell‐type‐specific therapeutic interventions.

In conclusion, a comprehensive “microbiota‐gene‐cell” framework was established that elucidates the causal relationship between GM and BC pathogenesis. Through rigorous multi‐omics integration, MCM6 and NR3C1 were identified as robust BC biomarkers with significant implications for diagnosis, prognosis, and targeted therapy. The links between these biomarkers and DNA replication pathways, immune microenvironment modulation, and epithelial cell differentiation were revealed, providing novel mechanistic insights into BC development. The identification of etoposide and specific traditional medicines as potential therapeutic agents offers promising avenues for drug repurposing in BC treatment.

This study has several limitations that should be acknowledged. Potential data heterogeneity from public databases may introduce selection bias despite rigorous MR design; the identification of only five protective taxa likely undersamples the ecological complexity of microbiome‐cancer interactions; and the prioritized biomarkers MCM6 and NR3C1 require experimental validation of their mechanistic roles in proliferation, DNA replication stress, and epithelial differentiation through in vitro/vivo models and clinical cohorts. In addition, single‐cell RNA sequencing is limited by a small sample size, particularly a single normal control, restricting robust interpretation and requiring validation in larger cohorts. These constraints underscore the imperative for prospective validation of causal taxa in diverse populations, mechanistic dissection of microbiome‐gene regulatory networks, and integration of host‐microbe co‐metabolomics to translate findings into targeted interventions. Future research should focus on validating these findings in larger clinical cohorts, investigating the subtype‐specific roles of these biomarkers, and developing targeted interventions based on the mechanistic insights provided by this study.

## Author Contributions

**Tianyu Luo:** writing – original draft, writing – review and editing, conceptualization, funding acquisition. **Mengyao Xue:** formal analysis, investigation, writing – original draft. **Yi Du:** writing – original draft, data curation, investigation, software. **Huiying Chen:** writing – original draft, data curation. **Ying Sun:** writing – review and editing, data curation. **Haidong Sun:** writing – review and editing, project administration, funding acquisition.

## Ethics Statement

The authors have nothing to report.

## Conflicts of Interest

The authors declare that the research was conducted in the absence of any commercial or financial relationships that could be construed as a potential conflict of interest.

## Policy on Using ChatGPT and Similar AI Tools

The author(s) declare that no Generative AI was used in the creation of this manuscript.

## Supporting information

Supporting File 1

Supporting File 2

Supporting File 3

Supporting File 4

Supporting File 5

Supporting File 6

Supporting File 7

Supporting File 8

Supporting File 9

## Data Availability

The datasets presented in this study can be found in online repositories. The names of the repository/repositories and accession number(s) can be found in the article/supporting material.
