# Supplementary figures and images for: The Mechanism of Gut Microbiota in Breast Cancer Based on the Bulk Transcriptome, Mendelian Randomization Analysis and Single Cell RNA Sequencing
Source: Microbiologyopen. 2026 Apr 10;15(2):e70284. doi: 10.1002/mbo3.70284 (PMC13067202; doi:10.1002/mbo3.70284)

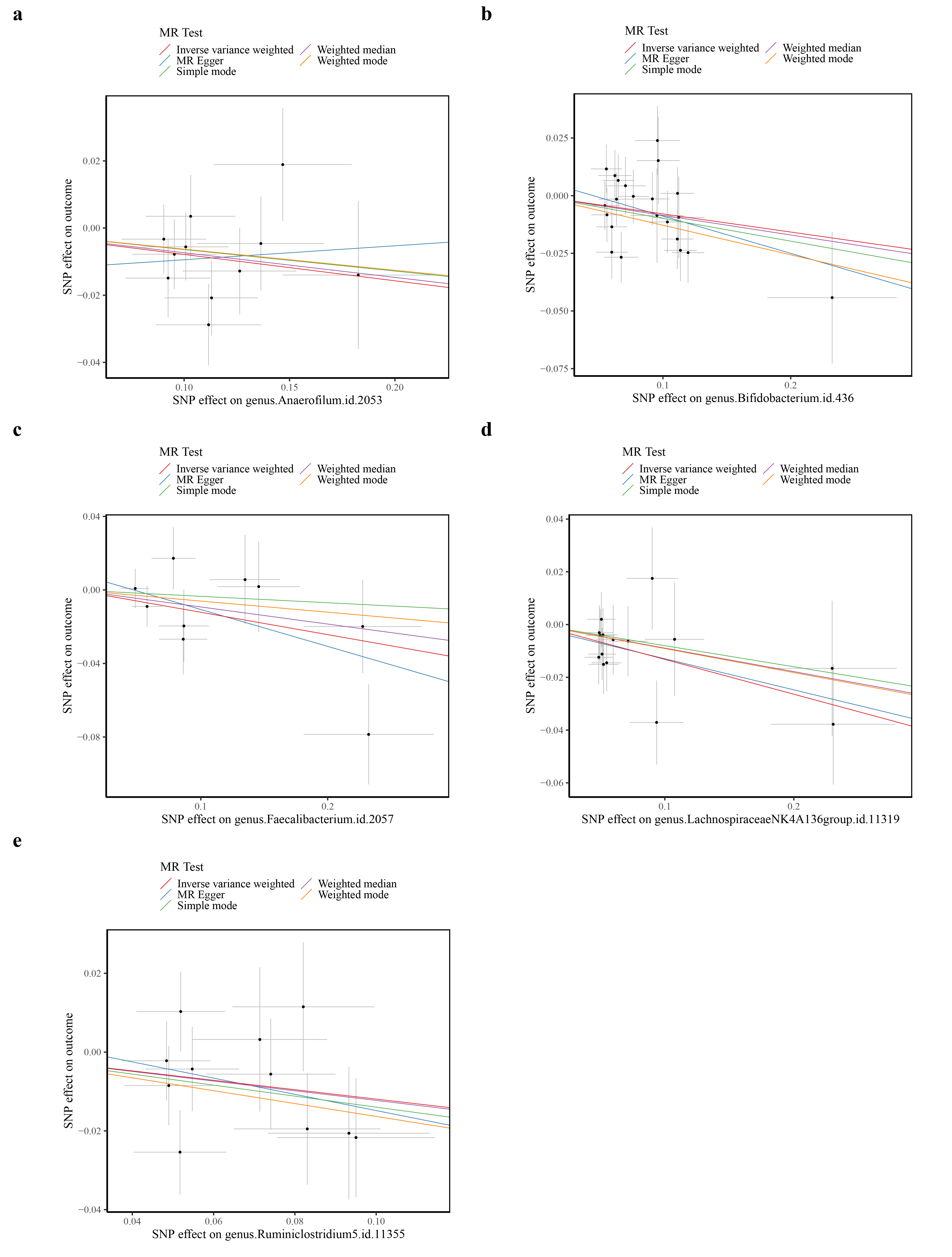

Supplement: Supplementary file 1 — Supporting File 1 [file MBO3-15-e70284-s007.tif]

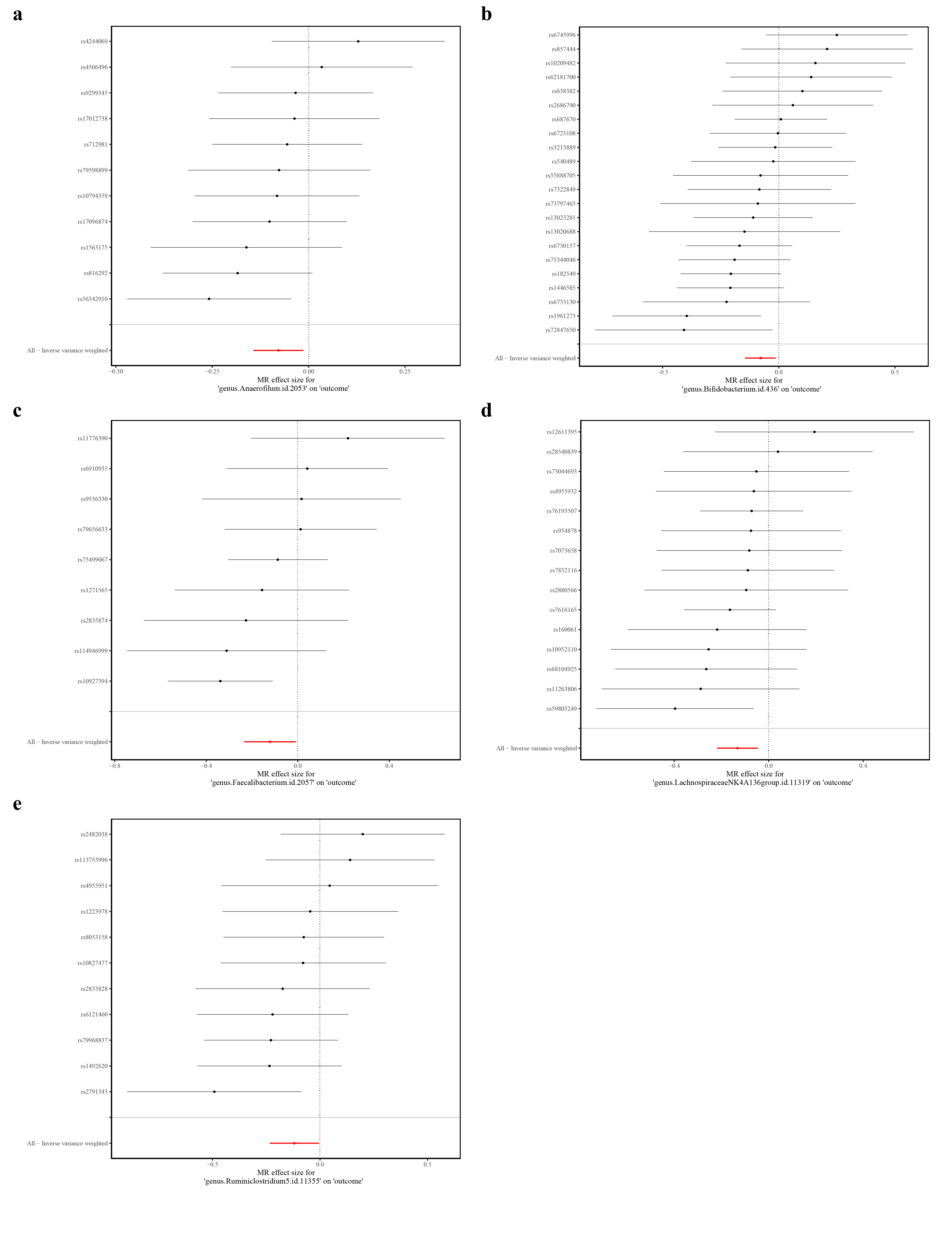

Supplement: Supplementary file 2 — Supporting File 2 [file MBO3-15-e70284-s008.tif]

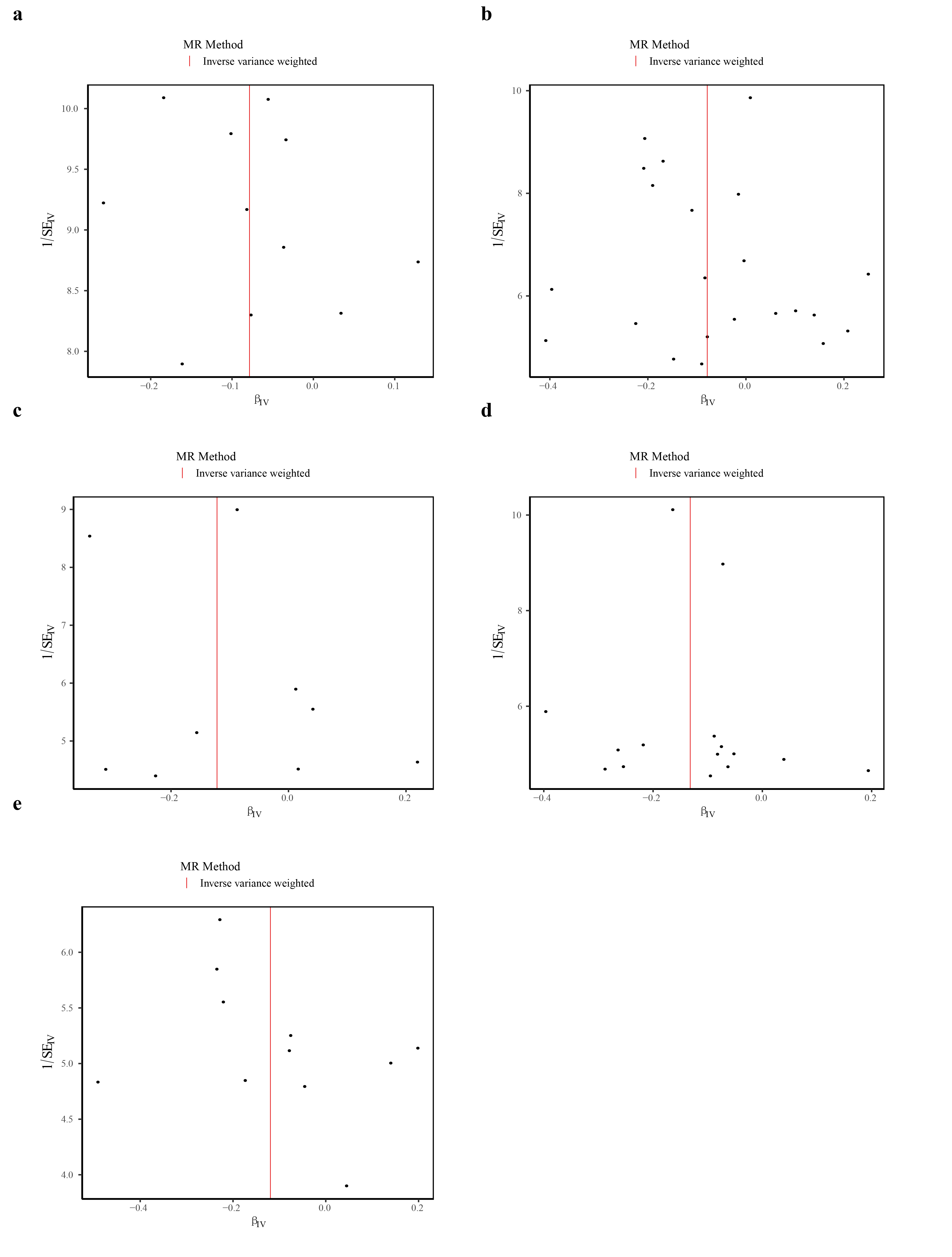

Supplement: Supplementary file 3 — Supporting File 3 [file MBO3-15-e70284-s002.tif]

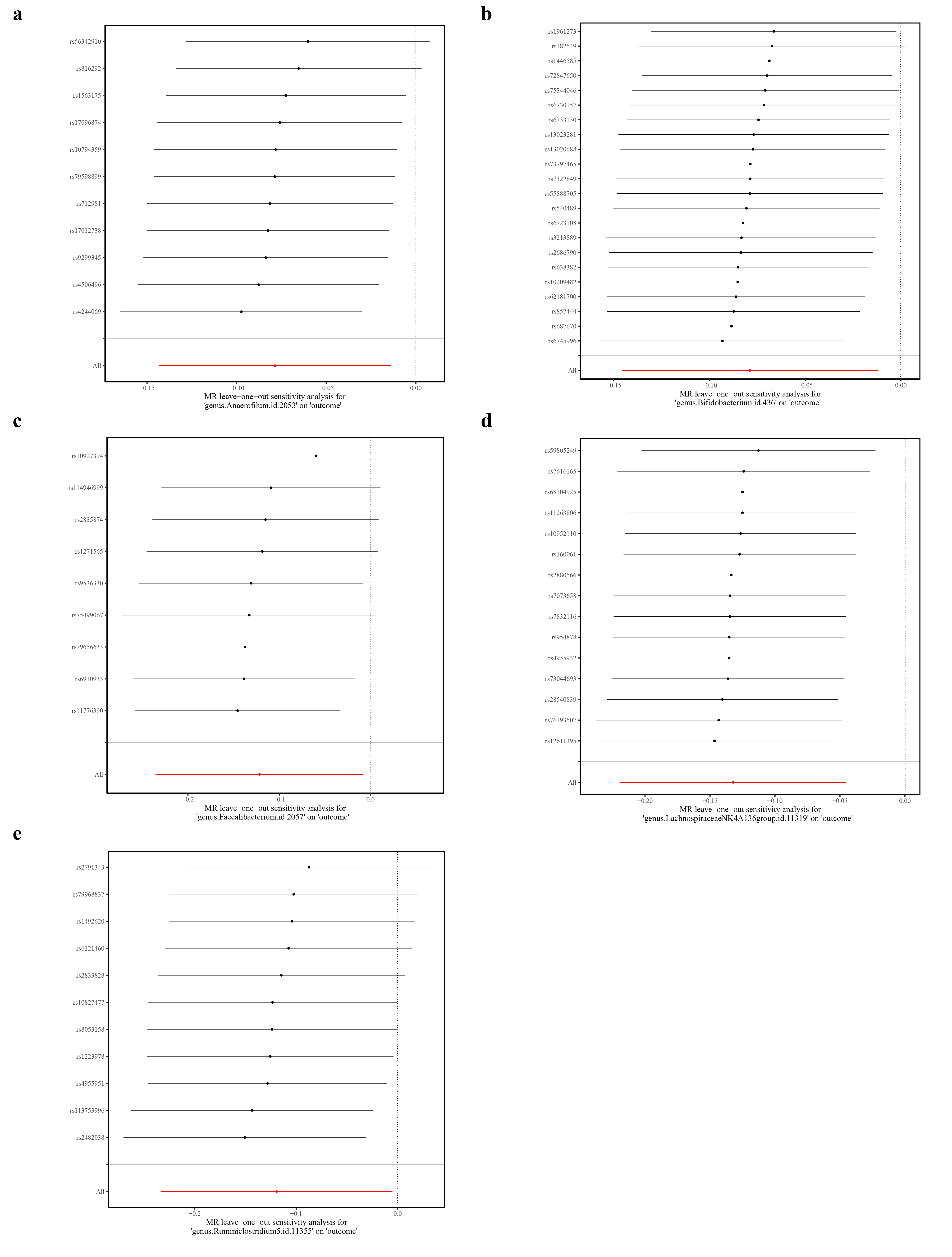

Supplement: Supplementary file 4 — Supporting File 4 [file MBO3-15-e70284-s003.tif]

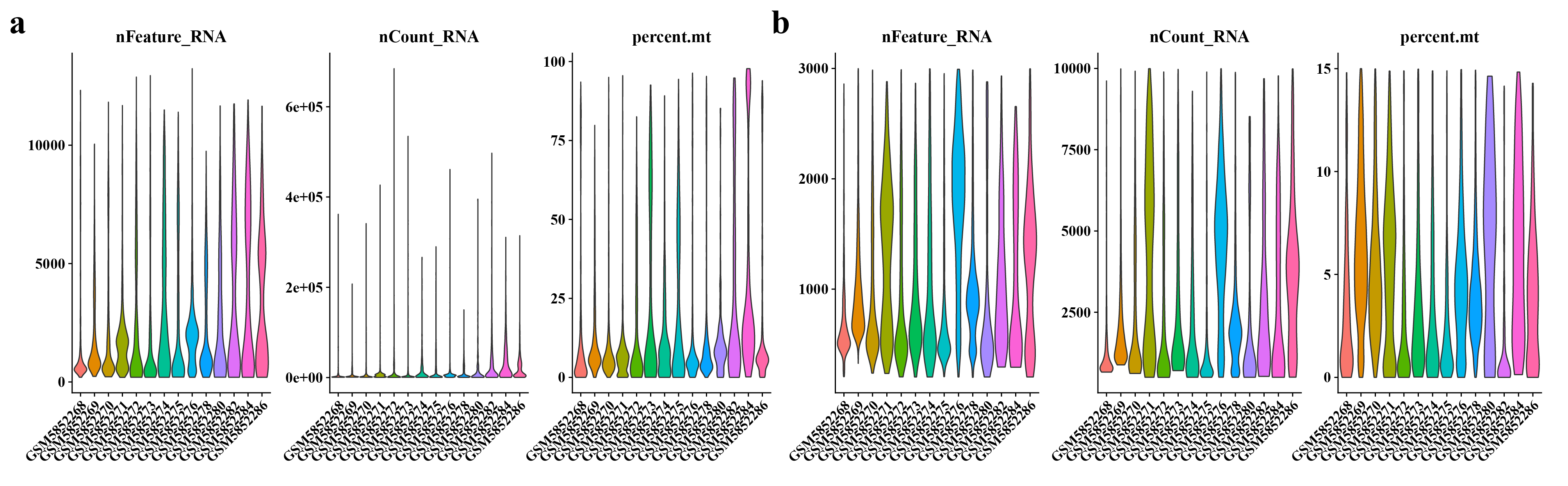

Supplement: Supplementary file 5 — Supporting File 5 [file MBO3-15-e70284-s009.tif]
